# Solid-State, Dye-Labeled DNA Detects Volatile Compounds in the Vapor Phase

**DOI:** 10.1371/journal.pbio.0060009

**Published:** 2008-01-22

**Authors:** Joel White, Kathleen Truesdell, Lloyd B Williams, Mary S AtKisson, John S Kauer

**Affiliations:** 1 Department of Neuroscience, Tufts University School of Medicine, Boston, Massachusetts, United States of America; 2 CogniScent, Inc., North Grafton, Massachusetts, United States of America; 3 Department of Biomedical Engineering, Tufts University, Medford, Massachusetts, United States of America; Monell Chemical Senses Center, United States of America

## Abstract

This paper demonstrates a previously unreported property of deoxyribonucleic acid—the ability of dye-labeled, solid-state DNA dried onto a surface to detect odors delivered in the vapor phase by changes in fluorescence. This property is useful for engineering systems to detect volatiles and provides a way for artificial sensors to emulate the way cross-reactive olfactory receptors respond to and encode single odorous compounds and mixtures. Recent studies show that the vertebrate olfactory receptor repertoire arises from an unusually large gene family and that the receptor types that have been tested so far show variable breadths of response. In designing biomimetic artificial noses, the challenge has been to generate a similarly large sensor repertoire that can be manufactured with exact chemical precision and reproducibility and that has the requisite combinatorial complexity to detect odors in the real world. Here we describe an approach for generating and screening large, diverse libraries of defined sensors using single-stranded, fluorescent dye–labeled DNA that has been dried onto a substrate and pulsed with brief exposures to different odors. These new solid-state DNA-based sensors are sensitive and show differential, sequence-dependent responses. Furthermore, we show that large DNA-based sensor libraries can be rapidly screened for odor response diversity using standard high-throughput microarray methods. These observations describe new properties of DNA and provide a generalized approach for producing explicitly tailored sensor arrays that can be rationally chosen for the detection of target volatiles with different chemical structures that include biologically derived odors, toxic chemicals, and explosives.

## Author Summary

Biological systems can provide engineering guidance on how evolution has solved particular problems. In the context of detecting chemicals in either the aqueous or vapor phase, two general biological approaches have emerged. The first relies on individual highly specific single receptors (sensors), each tuned to detect a single molecular species—examples include the receptors that mediate pheromone detection in insects or those that function in neurotransmission. Specificity is achieved by narrow band design. The second approach is implemented by arrays of receptors with relatively broad responses. In this case, specificity emerges from a constellation of receptor types that recognizes the molecule of interest—the canonical example here is the olfactory receptors in the main olfactory system of vertebrates. Specificity is achieved by a “one chemical–many broadly responsive detectors” paradigm. While trying to mimic the enormous odor coding ability of biological olfaction in an “artificial nose,” we searched for molecules with the requisite combinatorial capacity to serve as odor detectors. Here we show that single-stranded DNA molecules tagged with a fluorescent reporter and deposited onto solid surfaces can respond to vapor phase odor pulses in a sequence-selective manner. These findings demonstrate new properties of nucleotide molecules that can be exploited in engineered odor detection devices. In addition, this broadband responsivity to small molecules should be explored as a functional aspect of DNA (and RNA) as they exist in the normal cellular milieu.

## Abstract 3

Short sequences of solid-state DNA can selectively signal their interactions with small molecules in the vapor phase. These observations have been implemented in odor sensing in an electronic "nose" and further suggest that in vivo responses to small molecules may represent new, nongenetic attributes of DNA.

## Introduction

Odor sensor arrays composed of materials that are cross-reactive have advantages over narrowly tuned receptor systems. These include combinatorial responses to sets of compounds that exceed the number of receptor or sensor types, tolerance to partial system failure, and an ability to be flexibly trained, all of which are adaptive attributes that have emerged in biological systems over evolutionary time.

In building sensors for artificial noses, there are generally two strategies that are used to develop a diversity of detectors that is reminiscent of biological receptors and thus exploits the advantages of cross-reactive arrays. First is the explicit synthesis of polymers designed to interact with defined chemical properties of a target volatile compound (for example, fluorescent conjugated polymers designed to detect nitroaromatic explosives) [1–3]—these sensors are commonly designed to be highly specific. Second is the use of available off-the-shelf polymers to test for responses to target vapor phase compounds without prior knowledge of how their chemical properties generate different odor sensations. This second method has been used to make cross-reactive, polymer-based sensors in a number of array-based electronic nose devices, including the one we have developed [4–14]. Both methods have been effective for producing useful devices; however, the sensors tend to be labor intensive to build, yielding relatively small numbers of detector candidates. A more ideal class of sensing molecules would be one which, in addition to responding to volatiles, has the following properties: (1) has a chemistry that provides a large combinatorial complexity of structure, (2) has a molecular structure amenable to being replicated exactly in order to make large amounts of identical material, and (3) provides the opportunity to screen for sensors that can thereby be tailored to respond to many different volatile chemicals.

Sensors in artificial noses have not yet come close to achieving the sensor diversity and complexity found in biological olfactory systems [15]. As an alternative to the classes of synthetic polymers usually used for electronic nose sensors, we hypothesized that combinations of fluorescent dyes and a bio-polymer (namely, DNA) could be used as a sensing material. DNA is an attractive candidate for such use because of its stability and its potential for tremendous combinatorial complexity. Furthermore, once appropriate molecular sequences are identified, large numbers of identical DNA-based sensing molecules can be easily made using standard methods of synthesis. As described below, we have tested both double-stranded (dsDNA) and single-stranded DNA (ssDNA) using two methods of staining with fluorophores, and we report here the proof of concept demonstrating that ssDNA shows sequence-specific responses to a variety of volatile compounds and that libraries of these materials can be efficiently screened for odor responses. Portions of this work have been previously presented in abstract form [16–18].

## Results

### Responses Using dsDNA

**Figure 1:**
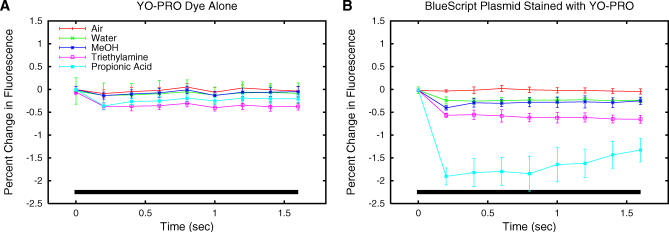
Changes in Fluorescence from Double-Stranded pBlueScriptSK DNA and YO-PRO Dye Sensors during Short Odor Sniffs The odor pulse began at 0 s and lasted for 1.6 s, indicated by horizontal black bar. (A) Responses of a sensor made from YO-PRO alone, then rinsed in 70% ethanol for 5 min. (B) Responses of a sensor made from YO-PRO and 5 ng total pBlueScriptSK DNA. Odor dilutions, expressed as fractions of saturated vapor, were: water, 10^−1^; methanol (MeOH), 10^−1^ (∼16,700 ppm); triethylamine, 10^−2^ (∼750 ppm); and propionic acid, 10^−1^ (∼390 ppm). Each trace represents the mean of 10 presentations; error bars indicate ± 1 SD.

**Figure 2:**
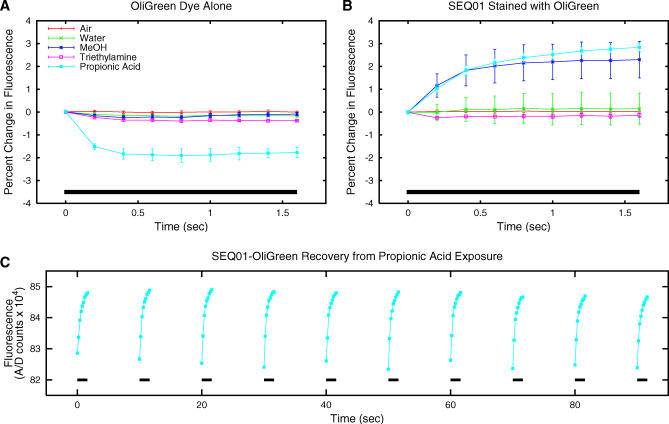
Changes in Fluorescence from ssDNA and OliGreen Dye Sensors during Short Odor Sniffs (A) Responses of a sensor made from OliGreen alone. (B) Responses of a sensor made from 20 μl of 10 μM oligomer SEQ01, stained with OliGreen. (C) Responses from SEQ01 to 10 repeated applications of 10^−1^ propionic acid (∼390 ppm), demonstrating return to baseline between sniffs. These 10 responses were used to calculate the mean shown in (B). See Figure 1 for details of odor presentation, odor dilutions, and description of data representation.

In an initial test of this hypothesis, we constructed sensors of dsDNA using a standard 2.9-kb pBlueScriptSK plasmid mixed with the intercalating DNA dye YO-PRO (Molecular Probes), dried onto a polyethylene substrate material, and tested in our electronic nose device [13,14] (see Materials and Methods). YO-PRO alone showed no changes in fluorescence over the time course of brief (1.6 s) odor sniffs (Figure 1A) applied by negative pressure in our artificial nose device. In contrast, sensors made from plasmid mixed with YO-PRO and dried onto the substrate produced large and rapid decreases in fluorescence upon exposure to propionic acid, with smaller or no changes when exposed to water, methanol, and triethylamine (Figure 1B). These odor responses were relatively stable over repeated trials (see Figure 2C for ssDNA). Responses from sensors made from dsDNA differing significantly in primary sequence (unpublished data), however, were qualitatively similar to those from sensors made from the pBlueScriptSK DNA (Figure 1B). We also constructed hairpin 33mer sequences that were much smaller than the plasmids described above and of about the size of the ssDNA described below, also stained with YO-PRO. These constructs of complementary G-C or A-T pairs (see sequences DS001 and DS002 in Table S1), which were designed to hybridize over a distance of 15 base pairs, all responded similarly, giving sequence-independent responses to the same odor set tested on the plasmid (unpublished data). These tests showed that, at least for these dsDNA constructs, sequence did not govern odor response.

### Responses using ssDNA

Because different dsDNA sequences did not show odor responses that were modified by changes in sequence, we then tested sensors made from short, ssDNA oligomers stained with the fluorescent dye OliGreen (Molecular Probes). OliGreen dye alone showed a decrease in fluorescence upon exposure to propionic acid, but little change to the other odors tested (Figure 2A). In contrast, SEQ01 (22 bases long; see sequence descriptions for SEQ01–SEQ30 in Table S1) stained with OliGreen and dried onto a polyethylene substrate had a markedly different odor response profile (Figure 2B) from OliGreen alone and from those using dsDNA and YO-PRO. This sensor sequence showed an increase in fluorescence in response to propionic acid and methanol (to which OliGreen alone did not respond), with relatively little change to the other odors tested. Furthermore, as shown in Figure 2C, the propionic acid signals from this sensing material returned to baseline levels between successive odor applications delivered as often as every 10 s, indicating that the odor–sensor interaction was reversible with a short time constant.

In these tests, OliGreen and YO-PRO were applied to the DNA in solution, prior to drying onto a substrate for testing. In applying the dyes in this way, there is little control over how and where the fluorophore binds to the DNA.

### Characterization of Response Diversity

To define better the dye–nucleotide interaction explicitly, we generated labeled oligonucleotides (20–24 bases long) by covalently attaching the fluorescent dye Cy3 (Amersham Biosciences) to the 5′ end during synthesis. We then adapted microarray techniques to screen these potential sensors for odor responses. An odor test chamber was constructed having the dimensions of a microscope slide to allow its use in a standard microarray scanner for examining the vapor responses from libraries of sensor sequences. Cy3-labeled oligonucleotides were spotted (∼50 μm diameter) onto cover slips, which were then mounted with the spots facing the interior volume of the test chamber (see Materials and Methods). Vapor-phase test odors were then injected into the chamber prior to scanning.

**Figure 3:**
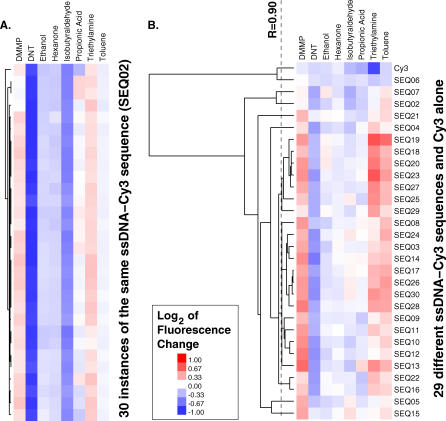
Odor Responses of DNA-Cy3 Sensor Spots Read with Microarray Scanner (A) Thirty SEQ02 control sensors (rows) tested with eight odors (columns). Pairwise Pearson correlation coefficients ranged from 0.91 to 1.00 (mean = 0.98, SD = 0.016). (B) Twenty nine different DNA-Cy3 sensors and Cy3 alone (rows) tested with the same odor test set as (A) (columns). Pairwise correlation coefficients ranged from −0.54 to 0.98 (mean = 0.66, SD = 0.32). Dashed line denotes correlation coefficient of 0.90. Data matrices show log_2_ transforms of fluorescence change between clean air and odor with graded red colors indicating the degree of fluorescence increase above baseline and blue indicating the degree of decrease. Dendrograms drawn to the same scale. Abbreviations: DMMP, dimethyl methylphosphonate; DNT, dinitrotoluene.

To measure odor responses using this method, we first tested control arrays in which the same DNA-Cy3 construct (SEQ02) was spotted at all locations. The responses of 30 replicates of the same SEQ02 construct (rows) to saturated vapors of eight odors (columns) are shown in Figure 3A (increases in fluorescence over baseline indicated by graded red colors and decreases indicated by graded blue colors). The responses of the replicated spots in this control array were highly correlated and therefore considered to be essentially identical. Pearson correlation coefficients calculated between pairs of sensors were all ≥0.90 (see Figure 3A, legend). This high degree of correlation is also represented by the compact cluster analysis dendrogram shown to the left of the data matrix in Figure 3B (see [19] for description of Pearson correlation coefficients and cluster analysis that are the standard methods applied to microarray data).

In contrast to the correlated responses from spots having the same sequence, odor response data from 29 different DNA-Cy3 sequences (Table S1) showed dramatic response differences (Figure 3B). Using a conservative correlation coefficient threshold of 0.90 (the minimum pairwise correlation from analysis of the SEQ02 control array), cluster analysis of these data indicates there are at least 10 discriminable sensor types in this small sensor library. Three clusters had Pearson correlation coefficients >0.90 (clusters to the right of the dashed line in Figure 3B) with others having much lower correlations. The response variety obtained with this small array is clearly sequence-dependent and strongly suggests that large numbers of different sensor types are likely to be found when larger numbers of DNA-Cy3 sensor candidates are tested. It is important to note that the sensors in this sample respond to a variety of compounds with different chemical structures and that all of the odor response profiles, with the possible exception of SEQ06, were distinctly different from that of Cy3 dye alone (Figure 3B, top row).

**Figure 4:**
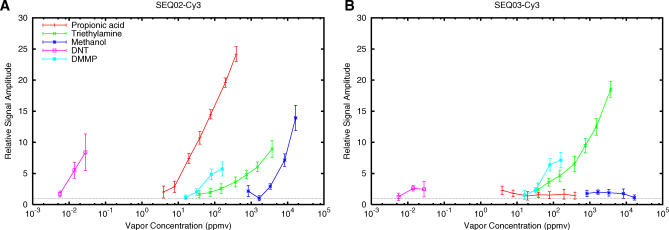
Odor Concentration-Response Curves Tested in the Artificial Nose for Two ssDNA Oligonucleotides Labeled with the Fluorescent Dye Cy3 during Synthesis (A) Responses from sequence SEQ02. (B) Responses from a different sequence SEQ03. Sensors were made from 20 μl of 10 μM oligomer. Each data point is the mean of 10 presentations; error bars indicate ± 1 SD.

In addition to response diversity, the sensitivities of a number of DNA-Cy3 sensors were also tested in our electronic nose device (similar to the tests shown in Figures 1 and 2) using an air-dilution olfactometer to deliver ranges of controlled odor concentrations. The dynamics of the responses of individual DNA-Cy3 sensors to sniffs of odorant were similar to those shown in Figure 2 for ssDNA stained with OliGreen. The concentration-response functions of different sequences for a small odorant test set summarize sensor concentration dependence as well as response diversity. For example, SEQ02 (Figure 4A) showed responses to propionic acid and triethylamine at lowest concentrations of 4 and 75 ppm, and to methanol, 2,4-dinitrotoluene (DNT; found in the vapor signature of TNT-containing landmines) [20–22], and dimethyl methylphosphonate (DMMP; precursor to Sarin nerve gas) at ∼33,900 ppm, ∼6 ppb, and ∼30 ppm, respectively. In contrast, SEQ03 (Figure 4B) responded to triethylamine at ∼75 ppm, to DMMP at ∼30 ppm, and showed no response to propionic acid, methanol, or DNT. The DNT response of SEQ02 (2 × 10^−2^ of DNT saturation is ∼6 ppb, or 2.3 × 10^−10^ M; see [23] for vapor pressure) indicates that these sensors are capable of detecting certain compounds with low vapor pressures at low concentrations. With the exception of polymers that are specifically synthesized for detecting nitroaromatic compounds [1,3], the DNA-based sensors described here are the only fluorescent polymeric sensor materials of which we are aware that show significant responses to DNT.

It should be noted that the responses used to plot Figure 4 consist of both positive- and negative-going changes in fluorescence, but the signs of these responses are not represented in these graphs, which are intended only to show the gradation of response over concentration ranges. It is also important to note that the responses of those sequences (e.g., SEQ02 and SEQ03) that have been tested both in the array scanner and in our electronic nose device show some differences, which can be due to the following: (1) the substrates onto which they were deposited (glass versus silkscreen); (2) the concentration of odor used for testing (saturated versus graded series with maximum concentration of 1/10 dilution); (3) the duration of odor exposure (an extended time of minutes versus 1.6 s); and (4) the buffers used for deposition. We know from other experiments that each of these variables influences response.

## Discussion

The solid-state, DNA-based vapor sensors described here have two key properties that are important for use in electronic noses: (1) diverse and broad odor response profiles, (Figure 3B) and (2) high sensitivity (Figure 4). These response properties, combined with the intrinsically combinatorial nature of DNA, indicate that using DNA in this way provides a major new approach toward designing multitudes of sensors for detecting and identifying volatile compounds. For example, using 21mers yields 4^21^ or more than a trillion different sequences. Further, these data describe a new property of dried, solid-state ssDNA—namely the ability to interact with vapor phase substances. The mechanisms underlying these responses are likely to be complex and we are currently in the process of trying to elucidate them. Briefly, the data we have so far indicate that fluorescence changes related to odor responses arise from minute changes in the three-dimensional structure of the dried ssDNA within the hydration shell that envelopes this hydroscopic molecule at normal relative humidities ranging from about 30%–70% [17,18].

It is clear that these solid-state, DNA-based odor sensors are distinctly different from other nucleotide-based sensing materials, such as aptamers. Although aptamers have been used to detect some kinds of small, non-nucleotide ligands, these interactions have all been carried out in aqueous solution [24–26], not with the oligomers dried onto surfaces, and they have not been used to detect vapor-phase compounds as described here. Furthermore, solid-state, DNA-based sensing materials for cross-reactive sensor arrays can be explicitly selected for nuanced differences in specificity and response breadth, whereas aptamers are selected for optimal “monospecificity”.

Analysis of sensor array and olfactory receptor properties that we have carried out using information theory [27,28] has shown that the breadth of cross-reactivity and the number of different sensor (or receptor) types are crucial parameters for describing how odor discrimination is carried out in both biological and electronic olfactory systems. Using the methods detailed here, sensors that are required to have a particular response breadth and sensitivity for a defined odor detection problem can be explicitly chosen from DNA libraries. Using standard microarray equipment and the odor test chamber described above, it is possible to rapidly screen thousands or hundreds of thousands of DNA-based sensors. A number of methods are available for generating large sensor libraries, including direct synthesis (e.g., lithographic [29] and ink-jet [30] methods) and PCR amplification of random libraries. It is therefore feasible to combine large-scale, DNA-based sensor library generation with high-throughput screening. This provides a means of selecting sensors appropriate for detecting volatile compounds related to many real-world problems in security, industrial quality control, environmental monitoring, and medical diagnosis [31–33].

Based on our early results published in abstract form [16], there have been two reports of DNA coated onto single-walled, carbon nanotube field effect transistors [34,35] in which sequences from our abstract [16] were used to detect vapor phase compounds. This method illustrates another way of potentially reading out odor-related responses from DNA.

## Materials and Methods

### Description of electronic nose.

Our electronic nose [13,14] uses an array of optically based chemical (polymer) sensors that change their fluorescence intensity upon exposure to brief pulses of vapor-phase compounds. The device contains 16 sensors that can be illuminated and observed at 16 different excitation and emission wavelengths. The sensors and the optical elements for illuminating and monitoring the sensors are placed along a narrow chamber through which target odors are drawn. Excitation light is produced by filtered LEDs providing bandpass wavelengths appropriate for the sensors being used (450 nm, bandwidth (BW) 40 for OliGreen and YO-PRO; 540 nm, BW 40 for Cy3). The fluorescent sensors emit light at longer wavelengths which passes through filters (550 nm, BW 70 for OliGreen and YO-PRO; 600 nm, BW 10 for Cy3) and is monitored by photodiodes. Thus, there is an LED/bandpass filter/sensor/bandpass filter/photodiode set for each sensor channel. Electric current produced by each photodiode is converted to voltage, amplified, and digitized (every 100 ms) over the time of the sniff duration (1–2 s) at nominal 20-bit resolution. The digital signals are recorded and processed by an embedded microprocessor. User control of the device is via a touch screen panel. The current device is self-contained, hand-held, weighs about 1.5 kg, and is about the size of a facial tissue box.

### Sensor tests in the electronic nose.

DNA was diluted to the desired concentration (0.2–50 ng/μl) in TE (10 mM Tris base, 0.5 mM EDTA, pH 8.0) or Tris-NaCl (2 mM Tris base, 11 mM NaCl, pH 8.0). Twenty μl of dilute DNA was mixed with 1 μl stock solutions of YO-PRO (1:40 in Tris-NaCl) or OliGreen (1:1 in Tris-NaCl) and incubated at room temperature for 5 min. Dye-only controls were made of 1 μl dye stock in 20 μl TE or Tris-NaCl. DNA/dye mixtures were applied to a substrate of acid-washed 16xx polyethylene silkscreen (10 mm × 12 mm) and allowed to dry for 25 min. Each sensor was rinsed in 70% ethanol for 5 min, allowed to dry, and then attached to supports on glass cover slips for testing in the electronic nose device. The dried DNA material adhered to the silkscreen mesh without occluding the openings. The exact thickness is unknown, but based on superficial appearance, it simply forms a thin film stuck to the strand supports that make up the silkscreen mesh. An air-dilution olfactometer of standard design and modeled after a system used in dog studies [36] was used to deliver controlled dilutions of odors to the electronic nose device. Odor dilutions were determined by the ratio of flow rates through the clean air and saturated odor channels. Total flow rate was 10 l/min.

### Sensor tests in the microarray.

Twenty-nine ssDNA sequences labeled with Cy3 on the 5′ end were synthesized using standard phosphoramidite chemistry. The constructs were reconstituted and diluted into buffer (10 mM Tris, 50 mM NaCl, pH 8.0) at a concentration of 4 μM. Sensor constructs were then spotted onto clean 22 × 60 mm cover slips using a BioRobotics MicroGrid II.

A chamber was constructed for testing sensor array responses to odors in a Packard ScanArray 4000 microarray scanner by milling a stainless steel blank to have the outer dimensions of a standard microscope slide (1 mm thick × 25 mm wide × 75 mm long), which the scanner will accept. A rectangular hole slightly smaller (20 mm × 57 mm) than a 22 × 60 mm cover slip was cut through the center of the blank, and shoulders were cut around the hole on each side to accommodate two 22 × 60 cover slips, which, when placed on the shoulders, created an interior volume of approximately 1 cc. Three edge holes were drilled from one end of the blank into the closed volume of the chamber formed by the cover slips in order to inject odors via a 22 gauge hypodermic needle. A blank cover slip was taped into the bottom shoulder, and a sensor array cover slip was taped into the top with the DNA spots facing the interior of the chamber.

For odor testing, 30-cc glass syringes containing KimWipes saturated with different chemical compounds (or containing crystalline solid, as in the case of DNT) were used to inject odor vapor into the test chamber immediately before scanning. Ten cc of vapor was injected into each of the three edge holes and allowed to escape through the remaining holes, leading to a 30-fold exchange of chamber air. After an odor test, the chamber was opened for 15 min to allow the odor to escape. Clean humidified air was injected before each odor test to maintain constant chamber humidity. For the data analysis shown in Figure 3, fluorescence changes are expressed relative to a clean air control presented in the same manner as an odor test. We used epifluorescence microscopy and video imaging to confirm that fluorescence changes in sensor spots after odor injection into the chamber were stable for at least two minutes, which is the time required for scanning the full sensor array. For the data shown in Figure 3, sensor spot fluorescence was quantified using Dapple (http://www.cs.wustl.edu/~jbuhler/research/dapple). Data were then analyzed using Cluster 3.0 (http://bonsai.ims.u-tokyo.ac.jp/~mdehoon/software/cluster/software.htm) and visualized using Java TreeView (http://jtreeview.sourceforge.net).

## Supporting Information

Table S1 The Sequences of the Two Double-Stranded (DSxxx) and 30 Single-Stranded (SEQxx) Molecules Tested (26 KB DOC)

## Abbreviations

BW: bandwidth
dsDNA: double-stranded DNA
ssDNA: single-stranded DNA
