# Supplementary material for: Solid-State, Dye-Labeled DNA Detects Volatile Compounds in the Vapor Phase
Source: PLoS Biol. 2008 Jan 22;6(1):e9. doi: 10.1371/journal.pbio.0060009 (PMC2211549; doi:10.1371/journal.pbio.0060009)
Supplement: Supplementary file 1 [file pbio.0060009.st001.doc]

**Supplementary Information**

Table S1

Label	Sequence

DS001	TTA ATA TAA ATT TTT CCC AAA AAT TTA TAT TAA

DS002	GGC CGC GCC CGG GGG TTT CCC CCG GGC GCG GCC

SEQ01	5’ Cy3-ACC AGG ACC TGA CTA AGC AGA T

SEQ02	5’ Cy3-GAG TCT GTG GAG GAG GTA GTC

SEQ03	5’ Cy3-CTT CTG TCT TGA TGT TTG TCA ACC

SEQ04	5’ Cy3-CTT CAT TCG AAA CCC GAA TG

SEQ05	5’ Cy3-ATC AGG TGG TTG ACC TGA TA

SEQ06	5’ Cy3-GGG GGG GGG GGG GGG GGG GGG

SEQ07	5’ Cy3-AAA AAA AAA AAA AAA AAA AAA

SEQ08	5’ Cy3-TTT TTT TTT TTT TTT TTT TTT

SEQ09	5’ Cy3-CCC CCC CCC CCC CCC CCC CCC

SEQ10	5’ Cy3-GTA CCG TCT ATA CTC TGA CCA

SEQ11	5’ Cy3-GAC ATC GGT GTC TGC GGG CAT

SEQ12	5’ Cy3-CCG ATC CAC ACA GAT CGC GGC

SEQ13	5’ Cy3-CTA TTA ATA GAC GCC CAC CAC

SEQ14	5’ Cy3-GAT TAA GTC GAG GTT ATC GGT

SEQ15	5’ Cy3-TCG TGG TAA ATC CCA CTG CTA

SEQ16	5’ Cy3-GGC AGC TCG TAA CGC GTG CGG

SEQ17	5’ Cy3-AGC GAT ACA TCG CGG ACC ACG

SEQ18	5’ Cy3-AGT CGA CAA TCG GTT TTC CTA

SEQ19	5’ Cy3-GAG AGT CTT CGG CAG GTA GCG

SEQ20	5’ Cy3-GCG AAT CTT ACT CTT CCA TAC

SEQ21	5’ Cy3-ACT TCA TCA GAG TGG GGG CAG

SEQ22	5’ Cy3-TAC TGG GCT AGC TGC GGA GGA

SEQ23	5’ Cy3-GAT TCG AAA TGG AGT ACC ACC

SEQ24	5’ Cy3-ATA ACG GTG TCT ACG CGT GAC

SEQ25	5’ Cy3-CAC TCC GAC CCA CGG GGC CCA

SEQ26	5’ Cy3-TGA AAG CCC TTT CGG TCT CCG

SEQ27	5’ Cy3-AAC CTT ATA CCT GGT GTT TGT

SEQ28	5’ Cy3-ACT GTC CAT TGG CAG GCC AGT

SEQ29	5’ Cy3-GGC ATT GGG ATG CTC CAA CTC

SEQ30	5’ Cy3-ATG TAA GAC TGT CTC CCC GTA
